# Recombinant Production and Characterization of a New Toxin from *Cryptops iheringi* Centipede Venom Revealed by Proteome and Transcriptome Analysis

**DOI:** 10.3390/toxins13120858

**Published:** 2021-12-02

**Authors:** Lhiri Hanna De Lucca Caetano, Milton Yutaka Nishiyama-Jr, Bianca de Carvalho Lins Fernandes Távora, Ursula Castro de Oliveira, Inácio de Loiola Meirelles Junqueira-de-Azevedo, Eliana L. Faquim-Mauro, Geraldo Santana Magalhães

**Affiliations:** 1 Laboratório de Imunopatologia, Instituto Butantan, São Paulo 05503-900, Brazil; lhiri.hanna@gmail.com (L.H.D.L.C.); bianca.tavora@butantan.gov.br (B.d.C.L.F.T.); eliana.faquim@butantan.gov.br (E.L.F.-M.); 2 Laboratório de Toxinologia Aplicada, Instituto Butantan, São Paulo 05503-900, Brazil; milton.nishiyama@butantan.gov.br (M.Y.N.-J.); ursula.oliveira@butantan.gov.br (U.C.d.O.); inacio.azevedo@butantan.gov.br (I.d.L.M.J.-d.-A.)

**Keywords:** *Cryptops iheringi*, centipede, venom, toxin, transcriptome, proteome, recombinant protein, venomics, chilopoda

## Abstract

Among the Chilopoda class of centipede, the *Cryptops* genus is one of the most associated with envenomation in humans in the metropolitan region of the state of São Paulo. To date, there is no study in the literature about the toxins present in its venom. Thus, in this work, a transcriptomic characterization of the *Cryptops iheringi* venom gland, as well as a proteomic analysis of its venom, were performed to obtain a toxin profile of this species. These methods indicated that 57.9% of the sequences showed to be putative toxins unknown in public databases; among them, we pointed out a novel putative toxin named Cryptoxin-1. The recombinant form of this new toxin was able to promote edema in mice footpads with massive neutrophils infiltration, linking this toxin to envenomation symptoms observed in accidents with humans. Our findings may elucidate the role of this toxin in the venom, as well as the possibility to explore other proteins found in this work.

## 1. Introduction

The Centipedes of the Chilopoda class are venomous arthropods that are widely distributed throughout the world [1,2,3]. The pair of glands, located in each jaw, produce venom that is used to kill or immobilize its prey by inoculation [4,5,6]. These animals are well adapted to urban areas and are commonly found in backyards and other home areas, and because of this, they often pose a danger to humans by injecting their venom as a defense [2]. The symptoms and complications induced by the envenomation caused by centipedes indicate that its venom comprises a natural set of proteins, peptides, and enzymes with a rich diversity of biological activities [7]. Most of the recent studies of the genus *Scolopendra* have indicated that the venom of a single centipede contains more than 500 proteins [8,9,10].

Centipedes’ venoms have been used for hundreds of years in traditional Chinese medicine, as well as in Korea and other countries in East Asia to treat many disorders such as stroke, hemiplegia, epilepsy, cough, tetanus, burns, cardiovascular diseases, and myocutaneous disease, among others [11,12]. These historical and ethnopharmacological practices indicate that these animals’ toxins could be explored for therapeutic uses and drug development. Despite this, the pharmacological properties of the toxins and the accidental envenomation of humans have not been studied extensively.

In Brazil, epidemiological data on accidents with centipedes are also very scarce. However, two retrospective studies that include occurrences recorded at the Vital Brazil Hospital of the Butantan Institute, São Paulo, Brazil, showed that the majority of accidents with centipedes were caused by the *Cryptops* and *Otostigmus* genus, with the first being responsible for more than 60% of the cases reported [2,13]. The envenomation symptoms are characterized by burning pain, paresthesia, edema, and local hemorrhage, and can develop into superficial necrosis [2,13,14]. A systemic reaction, although rare, may occur [15,16,17,18,19,20].

The toxicology of centipede venom has been understudied in Brazil, and the scarce literature that does exist generally refers to species of the Scolopendridae family, especially the genus *Scolopendra* [21,22,23]; this is mainly due to the difficulties of obtaining sufficient amounts of venom to conduct biological activities. In this context, the extraction of centipede venom can be time-consuming, and the yields are typically very low, even when it is extracted through electrostimulation [24].

To date, only Malta, et al. (2008) [25] have explored this class of venom in the literature, demonstrating nociception induction, edema, and myotoxicity in mice. However, this study was unable to further characterize the venom due to the difficulty of isolating the venom’s toxins. Therefore, the identification of proteins and peptides responsible for the symptoms in human envenomation is highly important for the development of better treatments. In addition, these molecules may have applications in toxinology, immunology, ecology, agriculture, and pharmacy. Thus, the present study, based on the transcriptome and proteome approaches, reports the gene expression profile of the venom gland, identifies novel toxins and characterizes a new toxin that has been named Cryptoxin-1.

## 2. Results

### 2.1. Identification of Toxins from Transcriptomic and Proteomic Analysis

In this study, we used a proteotranscriptomic approach to characterize the venom from *C. iheringi*, since no protein or gene sequence was available in public databases. Therefore, the venom was submitted for proteome analysis while the venom gland mRNA was extracted and submitted for transcriptome investigation.

The *C. iheringi*’s venom gland mRNA was extracted and sequenced by Illumina HiSeq 1500 technology (Figures S1 and S2 of the supplementary material). A total of 15,904,398 paired-end reads were generated. The relevant pre-processing quality control, filtering, and trimming steps were applied, resulting in 14,964,551 (94.1%) high-quality reads. The transcriptomic profile of the *C. iheringi* venom gland generated 88,774 assembled transcripts with an average length of 766 bp, a Transcript N50 of 1104 and contained 16,266 (18.3%) transcripts with a length of greater than 1 Kb (Table 1). We evaluated the completeness of the *C. iheringi* transcriptome assembly using BUSCO (Benchmarking Universal Single-Copy Orthologs), searching against the 954 metazoa ortholog groups, and identified 934 (97.8%) of the conserved groups in metazoa; of these, 885 (92.7% of total) were complete, and 49 (5.1%) genes were fragmented.

For the alignment of *C. iheringi* transcriptome assembly against the 106,197 transcripts from 10 species from the Scolopendromorpha orders (Table 2) (*C. anomalans, H. marginata, S. alternans, S. cingulata, S. dehaani, S. morsitans, S. subspinipes, S. virirdis, S. rubiginosus, S. sexspinosus*) we obtained a total of 5328 (6%) *C. iheringi* hits, with the *Cryptops anomalans* having the highest rate of identification, of 4272 (4.83%). The sequence similarity surveys, by BLASTx alignment, resulted in 71.4% of unknown transcripts. Therefore only 28.6% of all transcripts presented at least one protein homolog against the Uniprot and TSA databases.

To further characterize the toxins sequences, the crude venom was analyzed by LC-MS/MS, and then, we performed automatic peptide matching against the predicted proteins from the *C. ihering*’s transcriptome. The sequences identified by this approach were labeled as putative known toxins if they were present in a public database, and if not, they were referred to as putative unknown toxins.

Furthermore, the predicted proteins from the *C. ihering*’s transcriptome that were not identified in the approach above were labeled as non-toxins, aligned with the Gene Ontology (GO) database, and classified according to their main biological category, in accordance with the GO nomenclature. The remaining sequences that were not identified as a match within the searched databases, and which were not identified through the association between the transcriptome and total venom proteome, were called unknown transcripts and they were no longer explored.

**Figure 1:**
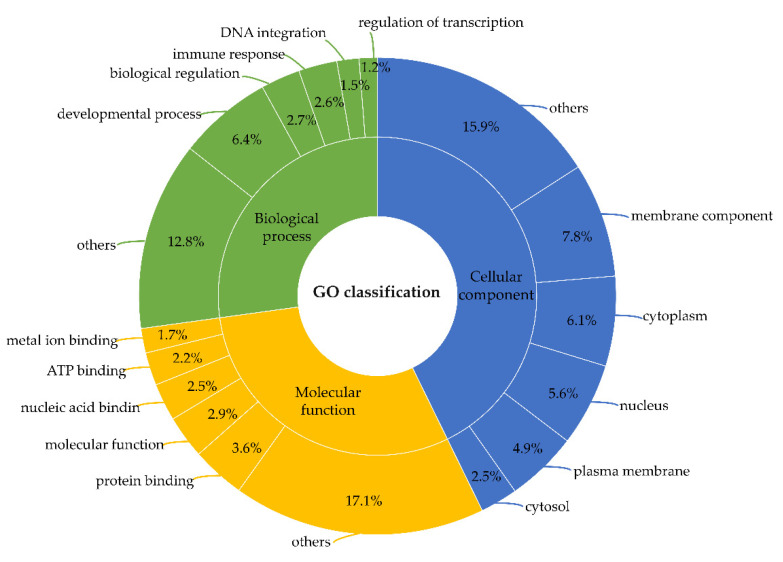
Non-toxins distribution of the five most representative categories of ontologies in the total number of transcripts from the transcriptome analysis of *C. ihering*’s venom gland. Annotation was performed according to the Gene Ontology terms for cellular component, biological process, and molecular function categories.

Among the non-toxins transcripts, around 6877.9 transcripts (27%) belong to the Biological Process, 10,953.7 transcripts (43%) to the Cellular Component, and 7642.12 transcripts (30%) to the Molecular Function, the five most representative categories for each GO term were represented as the percentage of transcripts in Figure 1.

**Figure 2:**
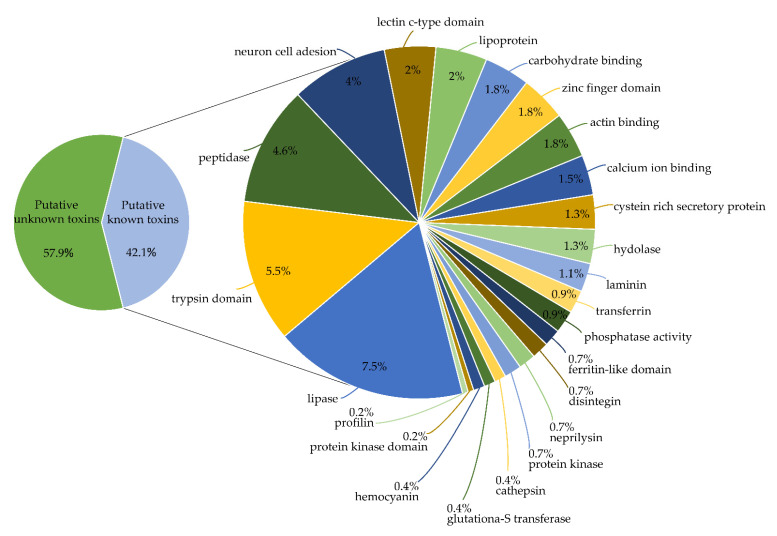
Distribution of the diversity of transcripts encoding putative known toxins found in the proteotranscriptomic approach of the venom gland of *C. iheringi*. Percentages correspond to the relative expression in TPM of each category.

The proteomic analysis of the crude venom revealed that 454 predicted proteins of the transcriptome could be classified as unknown venom components or as putative venom toxins, which were further classified into 24 different protein families (Figure 2). In terms of relative expression in TPM (transcript per million), putative unknown toxins and putative known toxins represented 24.97% of the transcriptome.

### 2.2. Novel Toxins Identified by the Mass Spectrometry

**Figure 3:**
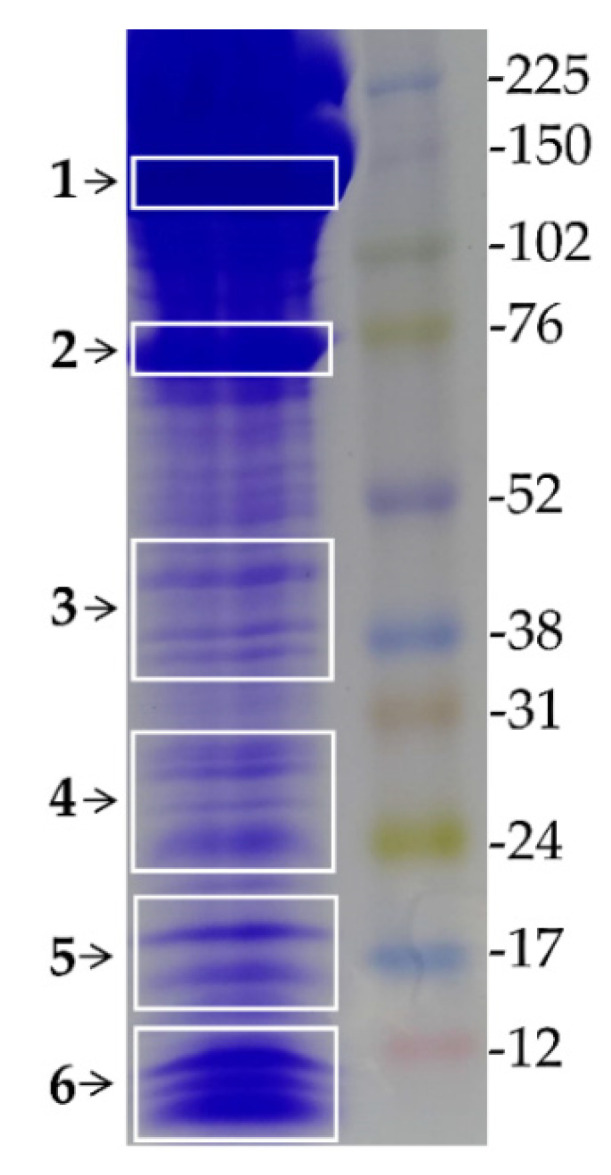
SDS-PAGE of the crude venom extracted from *C. iheringi*. Venom proteins were separated on a 12.5% SDS-PAGE gel and stained with Coomassie Brilliant Blue. The molecular mass (kDa) marker is shown on the right. The Selected groups of bands (from 1 to 6, on the left) were excised from the gel and processed for LC/MS-MS analysis.

In order to identify the major proteins of *C. ihering*’s venom, their approximate molecular masses, and relative abundances, the crude venom was submitted for an SDS-PAGE, followed by a mass spectrometry analysis of the selected gel bands. The results revealed bands with apparent molecular masses ranging from 15 to 200 kDa, with proteins of the high molecular mass showing a higher intensity (Figure 3).

Six groups of protein bands were selected and removed from the gel to be analyzed. The proteins were identified by matching the resulting mass spectra peptides to the deduced molecular masses of tryptic peptides, derived from full-length proteins as predicted from the transcriptome assembly. Matches of at least three peptides were considered valid. To identify a robust set of abundant venom toxins we focused on those sequences that exceeded the threshold value of 100 TPM. This allowed us to generate a list of 11 putative venom toxins that are highly abundant and likely play an important role in the venom (Table 3 and supplementary material Excel Table S1).

### 2.3. Cloning and Expression of Cryptoxin-1

After the venom proteotranscriptomic analysis, three putative unknown toxins that showed best proteome coverage (Ciheringi14246, Ciheringi38643, and Cryptoxin-1) were selected to be cloned and expressed in the recombinant form to study their biological activities. However, after initial evaluations, only the protein Cryptoxin-1 was obtained in a soluble form and with a satisfactory yield (8.5 mg/L of culture) after its expression in *E. coli*. This putative toxin, Cryptoxin-1, was characterized as described below.

**Figure 4:**
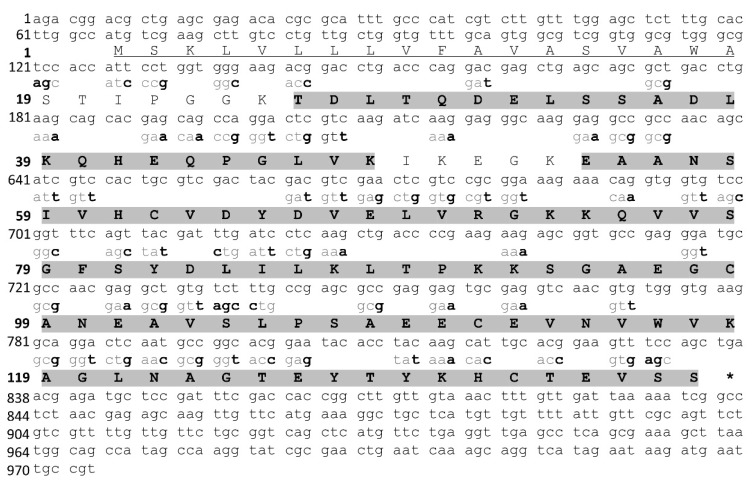
Nucleotide sequence of Cryptoxin-1 found in the transcriptome and its amino acid translation. The nucleotides in bold were changed to optimize expression in *E. coli*. The coverage of peptides found in the proteome is highlighted in grey. Underlined amino acids indicate the predicted signal peptide (SignalP-5.0), (*) indicates the stop codon.

Cryptoxin-1 is composed of 119 amino acids (Figure 4), with a predicted molecular mass of 12,769.33 Da, and a theoretical pI of 5.76. It also showed a predicted signal peptide, indicating that this toxin is secreted. In addition, it showed a GRAVY (Grand average of hydropathicity) index of −0.392, indicating its hydrophilic characteristics and the absence of a predicted glycosylation site.

**Figure 5:**
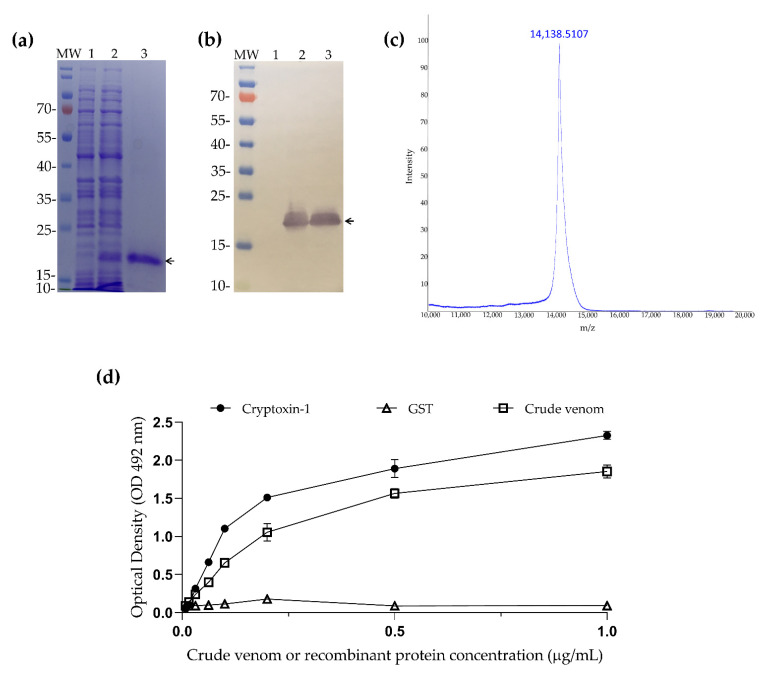
(**a**) 12% SDS-PAGE gel stained with Coomassie Brilliant blue. MW—Molecular mass marker in kDa; 1—*E. coli* sediment before IPTG induction; 2—*E. coli* sediment after IPTG induction; 3—Cryptoxin-1 purified by nickel-sepharose’s affinity. (**b**) Recognition of the recombinant protein by immunoblotting using the polyclonal antibody anti-histidine (Sigma–Aldrich, St. Louis, MO, USA). Arrow indicates protein height; (**c**) MALDI-TOF—Mass spectrometry analysis of purified Cryptoxin-1 showing its molecular mass of 14,138.51 Da. (**d**) ELISA, IgG anti—*C. iheringi* venom against crude venom, Cryptoxin-1, and GST (unrelated recombinant protein). Fixed antibody dilution used was 1:200 (7.5 ug/mL) versus serial protein dilution starting at 1 µg/mL.

The cDNA of Cryptoxin-1 was codon-optimized and cloned into the pET-24b (+) expression vector and then transformed into *E. coli* BL21 (DE3). The SDS-PAGE protein expression analysis revealed a single major band at around 16 kDa (Figure 5a, line 3). The mass spectrometry analysis (MALDI-TOF-MS) of purified Cryptoxin-1 showed a molecular mass of 14,138.5 Da (Figure 5c), which corresponds to the combination of Cryptoxin-1 (12,769.33 Da), a C-terminal tail of six histidines for IMAC purification, and additional residues encoded by the cloning vector (1360.67 Da). Its expression was also confirmed by polyclonal anti-histidine antibody immunoblotting, as shown in Figure 5b.

To further confirm the presence of Cryptoxin-1 in the venom, its recombinant form, as well as the whole venom and a recombinant non-related protein (negative control) glutathione protein S-Transferase (GST) from *Schistosoma mansoni* were tested by ELISA, using a purified polyclonal IgG anti- *C. ihering’*s venom. As can be seen in Figure 5d, the anti-venom IgG recognized both the venom and Cryptoxin-1, while the negative control (GST) was not recognized.

### 2.4. Crypotoxin-1 Induces Edema in Mice Footpad

As previously demonstrated, local tissue inflammation is one of the deleterious effects in *C. iheringi* envenomation [25]. Thus, we evaluated the local injury induced by Cryptoxin-1 injection in the footpad of BALB/c mice.

**Figure 6:**
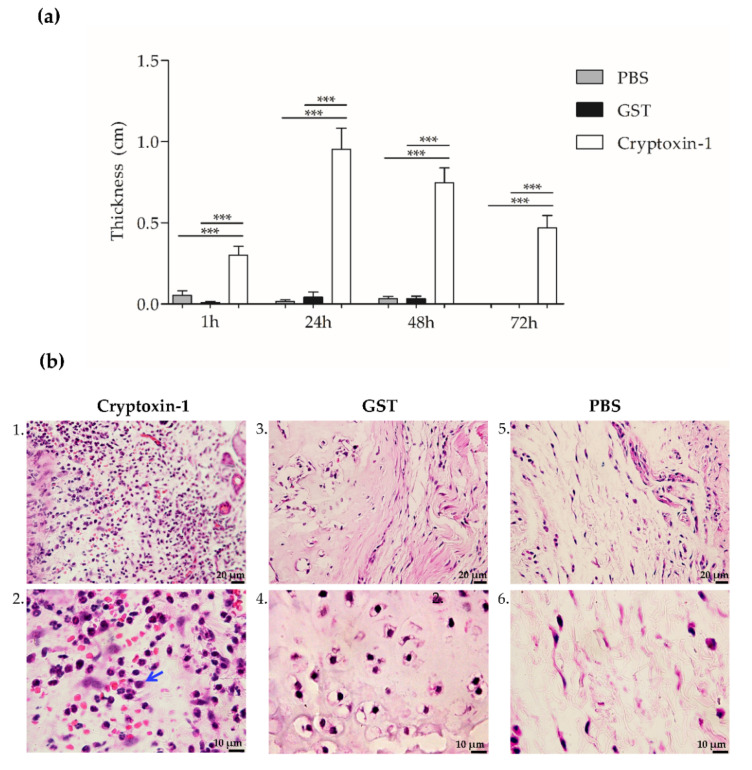
(**a**) Cryptoxin-1 induced footpad edema in mice. Groups of BALB/c mice were injected with 30 µL (45 µM) of Cryptoxin-1, GST (negative protein control), or 30 uL PBS (negative control). Edema was determined by thickness difference, at times 1, 24, 48, and 72 h. The results represent the ± S.E.M compared with the negative control group (Cryptoxin-1 vs PBS and Cryptoxin-1 vs GST), (*n* = 5). Statistical analysis was performed by ANOVA, followed by the Bonferroni test, *** *p* < 0.0001. (**b**) Histological analysis of the footpad of mice at 24 h after protein injection or PBS. All samples were analyzed with hematoxylin and eosin staining. 1. and 2.: Cryptoxin-1, bar 20 µm (40×) and 10 µm (100×) respectively; 3. and 4.: GST, bar 20 and 10 µm respectively; 5. and 6.: PBS, bar 20 and 10 µm, respectively. Neutrophilic inflammatory infiltrates (arrow). The images are representative of five mice/groups.

Mice were injected, through the right footpad, with either PBS (negative control), 45 µM of recombinant proteins Cyptoxin-1, or GST (negative protein control). The edema was measured through the thickness of the footpad at different time intervals, including: 1, 24, 48, and 72 h. The group injected with Cryptoxin-1 experienced a marked presence of edema during all the measurement times with a statistical difference when compared to the control groups (Figure 6a).

The cellular infiltration was then analyzed using the histological sections. Twenty-four hours after the injection, the GST and PBS groups presented normal tissue without an excess of inflammatory infiltration (Figure 6b, images 3, 4, 5, and 6). In contrast, 24 h after the Cryptoxin-1 injection, we observed the predominance of neutrophilic inflammatory infiltration (Figure 6b, images 1. and 2.).

### 2.5. Cryptoxin-1 Induces Potent Neutrophil Migration in Mice Footpad

**Figure 7:**
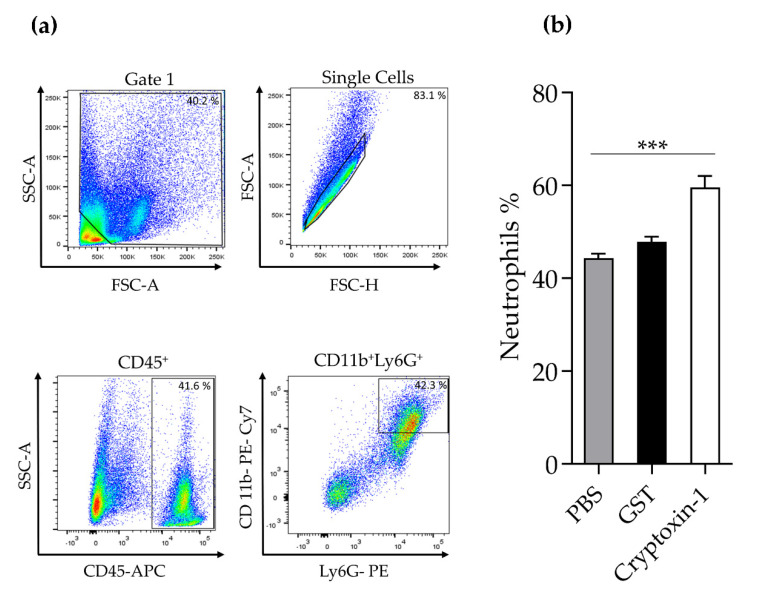
Neutrophil migration in the footpad of BALB/c mice injected with Cryptoxin-1, GST (45 µM), or PBS. (**a**) Flow cytometry gate strategy. Cells suspensions were prepared from footpad macerates after 24 h of the injection. Samples of cells (1 × 10^6^ cells) were incubated with anti-CD 45- APC, anti-CD 11b-PE-Cy7, and anti-Ly6G (PE) antibodies followed by flow cytometry analysis. (**b**) The mean of the percentage of CD45^+^CD11b^+^Ly6G^+^ cells of individual mice/group (*n* = 5) ± S.E.M. Statistical analyses was performed by ANOVA, followed by Bonferroni test, *** *p* < 0.05 Cryptoxin-1 group compared with PBS or GST groups.

Since we verified the peak of the edema induced by Cryptoxin-1 injection, as well as neutrophil infiltration in the histological analysis at 24 h, we confirmed this cellular profile by flow cytometry. Thus, at the peak of the edema (24 h), cellular suspensions were prepared from the footpad of the different mice groups and stained with anti-CD45, anti-CD11b, and anti-Ly6G mAbs conjugated to fluorochromes followed by flow cytometry. As shown in Figure 7, Cryptoxin-1 induced a significant level of neutrophils infiltration compared to that achieved in the other groups.

## 3. Discussion

Centipedes are well adapted to urban areas and are very commonly found in gardens and other residential areas. As a consequence, there is a great risk of accidents occurring for humans [2,13,26]. Although their venom may cause undesirable effects, centipedes have been used in traditional eastern medicine for centuries [11]. However, individual substances have rarely been refined [27,28].

The vast majority of studies of centipede venom are restricted to the *Scolopendra* genus [8,9,10,27,29,30,31,32,33]. In addition, some studies use the whole centipede instead of the whole venom for their proteomics analyses, making a more specified comparison inviable [34,35]. Five comparative studies have demonstrated that centipede venoms are complex cocktails, encompassing more than 60 phylogenetically distinct protein families [10,32,36,37,38]. Among them, there exist, enzymes, protease inhibitors, a great diversity of cysteine-rich proteins, and unknown proteins that are yet to be functionally characterized. Therefore, in this study, we aimed to contribute to the understanding of the toxin genes present in centipedes by generating a gene expression profile of the venom gland of *Cryptops iheringi* species.

Since literature for this species is scarce, we followed the transcriptome and proteomic approaches that were effective to identify toxins for other related species. In this regard, Ward, et al. (2018) [33], using these techniques, were able to identify 39 new toxins in the venom gland of the *Scolopendra viridis*, while Liu, et al. (2020) [8] found more than 400 toxin-like unknown sequences in the venom gland of *Scolopendra mojiangica.* Similarly, we found as high as 57.9% of the proteins to be uncharacterized from the *C. iheringi* centipede and 454 protein sequences that could only be characterized as putative unknown toxins or known toxins due to the proteomic approach. Among them, 263 proteins showed no similarity with the available sequences in public databases, indicating a great diversity of components with an unknown structure and function.

The putative venom toxins of *C. iheringi* revealed diversely distributed proteins with novel structures and biological activities that need to be further investigated. The majority of the venom proteins are putatively functional enzymes. Most notably, lipases and other hydrolases (8.8%), which include a large group of different proteins, such as phospholipases, are frequently reported as venom components of several other arthropods, such as centipedes, spiders, and scorpions [25,34,39,40,41,42,43], contributing to prey digestion and venom toxicity [42].

Trypsin domain proteins were also found in this venom (5.8%). Food protein degradation is crucial for digestion and is catalyzed by trypsin enzymes. Trypsin appeared early in evolution, and it became the most abundant proteinase in the digestive systems of invertebrates [44]. Trypsin performs two main functions, namely, the hydrolysis of protein and the activation of other digestive proteases, although it also plays a role in the innate immunity of these animals [45]. Some trypsin domains proteins have also been found in the centipede *S. subspinipes dehaani* venom gland transcriptome [29].

Peptidases (4.6%) were found to be another relevant group, comprised of endopeptidases, carboxypeptidases, and esterases that are among the reported protein components of some centipedes [30]. These kinds of proteins have an effect on amino acid production for digestive purposes and may be responsible for the tissue deleterious effects of the envenomation [46]. Several classes of peptidases, for which activities were not yet clarified, have also been found in the venom proteome and transcriptome of the scorpion *Hadrurus spadix* [47].

Putative neuron cell adhesion toxins were also present in this venom (4%). Findings on black widow spider venom indicate that such toxins can modulate a neuronal adhesion receptor, which stimulates strong neuronal exocytosis in vertebrates, and, interestingly, may perform functions in synapse development [48,49]. In the context of venom activity, interesting studies exist showing that a toxin from the snakes *Bothrops atrox* and *Bothrops moojeni* is capable of improving spatial memory disorder in temporal ischemic rats through its effects on the neural cell adhesion molecule [50]. Regarding the other putative venom toxin found here, more studies are necessary to propose and define its function in the venom of arthropods, especially from *C. iheringi*.

In addition to the whole venom proteome, *C. ihering’*s crude venom was subjected to a protein separation by SDS-PAGE to better visualize the main bands and their relative expression. In this gel, the venom showed an electrophoretic profile with a wide range of proteins between 15 and 200 kDa, with a large amount located above 70 kDa. The main bands of the venom were excised from the gel and subjected to LC-MS/MS mass spectrometry, in a strategy successfully utilized for the proteome decomplexation of other venoms [51]. The proteomic analysis returned several peptides with good spectra quality, which allowed us to classify 11 toxins, five of which ranged in size between 17 and 37 kDa, whose sequences did not show any similarity to the public databases, and therefore, represent new *C. iheringi* specific toxins. In order to unravel the function of unknown putative toxins, one, named Cryptoxin-1, was cloned and expressed in *E. coli.* The presence of this toxin in the venom was further confirmed by ELISA, where it was strongly recognized by IgG anti- *C. iheringi*’s venom, indicating its presence in the venom.

As it is known from the literature, envenomation by centipedes usually causes pain, erythema, and edema formation in humans and mice [13]. However, the characterization of the inflammatory activities induced by the venom is poorly described in the literature. For the centipede *C. iheringi*, there is only one published article showing that the venom induces strong pro-inflammatory activity able to induce edema and nociception, in addition to being myotoxic for mice [25]. Similarly, previous studies showed that the crude venom of the centipede species *S. viridicornis*, and *O. pradoi* induced edema in mice’s footpads, which progressively diminished by 72 h [25,52]. In this respect, the injection of Cryptoxin-1 into mice’s footpads was able to cause an edema of rapid evolution and progressive decay after 72 h. In addition, the injected animals were prostrate, bristly, with low temperature, and showed erythema at the injection site (data not shown).

After considering the results obtained with the edematogenic activity, we performed a histological analysis of the mice footpad injected with Cryptoxin-1 to characterize the cellular influx in the peak of the edema. The histological sections demonstrated the predominance of neutrophil infiltration, and flow cytometry analysis confirmed this result. Following these findings, Fung et al. (2011) [26] reported that 40% of patients who have been admitted to Hong Kong Emergency Hospital with centipede bites (species not specified), showed an increased neutrophil-predominant leukocytosis in their blood tests with an edema and erythema at the bite site, and strong pain. We also observed that the neutrophil infiltration lasted up to 72 h, which was also reported for the crude venom of *S. viridicornis* [53]. Taking these observations together, the results indicate that Cryptoxin-1 may contribute to the symptoms observed in envenomation. It is important to point out that in all the experiments, the recombinant GST, which was subjected to the same expression and purification procedures as Cryptoxin-1, was used as a non-related protein control to exclude any effect related to the protein purification steps.

Kinetics cellular infiltrate studies show that neutrophils are the first inflammatory cells to reach the lesion site and that edematogenic activity may occur due to the neutrophils release of cytokines, prostaglandin, myeloperoxidase, bradykinin, and histamine, causing increased vasodilation and the permeability of small vessels, resulting in the migration of other cells to the local tissue [54,55]. Although it is already known that innate immune cells participate in the local inflammatory response [27], the correlation between the local edema induced by *C. iheringi* venom and cellular infiltration is not completely understood. Therefore, further investigation is necessary to elucidate the complex interplay of the toxins present in its venom.

In this work, we described the profile of toxins present in the *C. iheringi* venom gland using transcriptome and proteome approaches that may contribute to understanding the venom composition and its effects in envenomation. In addition, new toxin genes were identified that may allow for the characterization of their role in this venom, and possibly for other toxins in related species. Furthermore, a new recombinant toxin named Cryptoxin-1 was also characterized as showing a proinflammatory activity, suggesting that it is likely to be one of the components responsible for the envenomation symptoms observed in accidents with humans. Additional studies are being conducted with this toxin as well as the other unknown toxins to understand their role in envenomation. Keeping this in mind, we understand the potential of novel developments for further studies concerning this centipede species and its venom.

## 4. Materials and Methods

### 4.1. Specimen Collection and Venom Extraction

Seven *C. iheringi* adult specimens were collected in the metropolitan area of the city of São Paulo, Brazil with the permission of SISBIO (15222-2) and kept in the Arthropod Laboratory of the Butantan Institute. To obtain the venom, the animals were anesthetized by anoxia, and the venom was extracted through electrical discharges (12 V) in the ventral region of the head (coxo sternum) with an electroshock device. The venom obtained through the forcipules was aspirated with an automatic micropipette and deposited in a microcentrifuge tube in an ice bath. The venom obtained was stored at −80 °C for a subsequent proteome analysis. The extraction was performed every 30 days.

### 4.2. RNA Isolation, Library Preparation, and Illumina Sequencing

The heads of seven specimens of *Cryptops iheringi* were submitted for the dissection the of venom glands for transcriptomics. The total RNA was extracted with TRIZOL Reagent (Invitrogen, Life Technologies Corp., Carlsbad, CA, USA), a method based on the procedure described by Chomczynski et al. (1987) [56]. The total RNA was quantified by its absorbance at a wavelength of 260 nm in a NanoDrop 2000 device (Thermo Fisher Scientific, Waltham, MA, USA). Beginning with an amount of total RNA ranging from 75 to 77 µg for each sample, the purification of mRNA was performed through an affinity to magnetic microspheres containing oligo (dT), using the protocol of the Dynabeads^®^ mRNA DIRECT kit (Invitrogen, Life Technologies Corp.), with reagents to reduce the number of ribosomal RNA (rRNA). The quantification of mRNA was performed using the Quant-iT RiboGreen^®^ reagent (Invitrogen, Life Technologies Corp.), according to the manufacturer’s specifications. All RNA procedures were performed using RNAse-free tubes and tips with a filter and water, treated with diethylpyrocarbonate (DEPC, Sigma–Aldrich, St. Louis, MO, USA). After the extraction of mRNA, its integrity was assessed using the 2100 Bioanalyzer, pico chip series (Agilent Technologies Inc. Santa Clara, CA, USA). The mRNA was then subjected to a purification and concentration step using the MinElute^®^ PCR Purification Kit (Qiagen) protocol. To confirm that mRNA was not lost of during this purification and concentration step, a further quantification of the mRNA was performed through its absorbance at a wavelength of 260 nm in a NanoDrop 2000 device (Thermo Fisher Scientific, Waltham, MA, USA).

A cDNA library was generated by TruSeq RNA Sample Prep Kit protocol (Illumina, San Diego, CA, USA). The cDNA was synthesized from fragmented mRNA using random hexamer primers, followed by ligation with appropriate sequencing adaptors. The size distribution of the cDNA libraries was measured with a 2100 Bioanalyzer using DNA1000 assay (Agilent Technologies Inc. Santa Clara, CA, USA). An ABI StepOnePlus Real-Time PCR System with *KAPA* Library *Quantification* was used for library sample quantification before sequencing. The cDNA library was then sequenced on Illumina HiSeq 1500 System, in a Rapid Run mode in a 2-lane paired-end flowcell, run for 300 cycles, generating 2 × 151 bp paired-end reads for each fragment, according to the manufacturer’s protocol (Illumina).

### 4.3. RNA-Seq Raw Data Pre-Processing, De Novo Assembly, and Functional Annotation

After large-scale sequencing of the cDNA, using Illumina HiSeq1500 equipment, bioinformatics analyses were performed. Thus, the sequencing platform generated sequencing images, which were converted to BCL format, after the CASAVA software was used to demultiplex the samples through the identification of the indexes (barcodes). The demultiplexing step generates the FASTQ file format, with a quality control of Q30.

For the pre-processing of the reads, an in-house pipeline was used to analyze the raw reads with a read filter by quality, eliminating reads with homopolymer and low complexity regions, poly-A/T/N tails, removal of adapters, indexes, and low-quality edges using the software FASTQ-mcf, [57] and bowtie2 [58]. The criteria used for filtering were as follows: the removal of homopolymer regions and a low complexity above 90% of the sequence, trimming tip regions with an average quality lower than 25. Only reads at a minimum size of 40 bp were kept. The raw reads were filtered by PhiX contaminants using the software Bowtie2 [58] standard parameters.

The transcriptome was assembled using the rnaSPAdes [59] with a K-mer size of 55.

The TransDecoder software version 3.0.1 (http://transdecoder.sourceforge.net/; accessed on 15 January 2018) was used to identify Open Reading Frames (ORFs) from the assembled transcripts with protein lengths higher than 60 amino acids. The program SignalP version 5.0 [60] was used for signal peptide predictions.

The completeness of the transcriptome was also estimated by the presence of sequences belonging to the set of ultraconserved eukaryotic proteins, tested using the BUSCO approach based on metazoa database [61].

Using TSA/NCBI, we downloaded the transcriptome assemblies from 10 species from the Scolopendromorpha orders (Table 2) (*Cryptops anomalans* (GERT01.1), *Hemiscolopendra marginata* (GHBY01.1), *Scolopendra alternans* (GASK01.1), *Scolopendra cingulate* (GCAP01.1), *Scolopendra dehaani* (GBIM01.1), *Scolopendra morsitans* (GHKQ01.1), *Scolopendra subspinipes* (GGDW01.1), *Scolopendra virirdis* (GGNE01.1, *Scolopocryptops rubiginosus* (GCIY01.1), *Scolopocryptops sexspinosus* (GHBZ01.1)) summarizing 106197 transcripts used to create the database for Blast alignment. The C. iheringi were aligned against the Scolopendromorpha database using the BlastN alignment tool with a cutoff of 1 × 10^−15^.

The predicted amino acid sequences were aligned using the BLASTx and BLASTp programs [62] against NCBI’s Uniprot/Swissprot protein databases, and Transcriptome Shotgun Assembly (TSA), to access sequence similarity with proteins in other species with a cutoff e-value of 1 × 10^−5^. The hmm search tool [63] allowed us to identify the conserved PFAM domains [64], with a cut-off e-value < 1 × 10^−3^. The priority order of the UniProt/Swissprot, PFAM, and TSA-NCBI protein hits was used to select the best candidate for each transcript.

The sequencing reads were aligned against the *C. iheringi* transcriptome with the bowtie2 program [58]. The method was used to estimate the transcript abundance. Further computing of the abundance for each transcript was performed by RSEM [65], along with a Maximum Likelihood abundance estimate, using the Expectation-Maximization algorithm for its statistical model. Final abundance estimates were calculated as Expected counts, Fragments Per Kilobase of exons per Million fragments mapped (FPKM) and Transcripts Per Million (TPM) values. Functional annotation was performed using the Blast2GO program [66], which is a tool used for analyzing a set of sequencing tags that makes it possible to understand the physiological meaning of a large number of genes. Transcript sequences were used as input sequences for the Blast2GO program. BLASTx was used to find counterparts in the NCBI database NR with a cut-off value of 1 × 10^−5^. Furthermore, the analysis was performed using the first 20 hits, a minimum alignment length of 33 amino acids, and a low complexity filter activation. The program then extracted the Gene Ontology (GO) terms for each hit obtained by mapping the existing annotation associations, after an annotation rule assigns the GO term to the sequence in question. After the BLAST, mapping, and annotation steps, the graphs, tables, and organization charts provided by the program were analyzed. For the distribution data of the GO terms provided by the program, tables with raw data were used instead of the graphs provided, since this allowed for greater formatting flexibility for the presentation of the data.

Bioinformatics analyses were performed using the computational infrastructure of the Center of Toxin, Immune response and cell signaling (CeTICS), and the Bioinformatics and Computational Biology Core in the Butantan Institute. The raw data generated in this project was deposited in the NCBI BioProject section under the accession code PRJNA763193, BioSample SAMN21432369 and SRA SRR1608688.This Transcriptome Shotgun Assembly was deposited in NCBI TSA under the accession GJOG00000000.

### 4.4. SDS-PAGE and LC-MS/MS Analysis of C. iheringi

*C. iheringi* venom was analyzed by SDS-PAGE (12% acrylamide resolution and Pierce, USA) under reducing conditions [67]. After protein separation by electrophoresis, the gels were stained with Coomassie Brilliant Blue R-250 (GE Healthcare) (0.1% coomassie R-250, 40% ethanol; 10% acetic acid) and bleached with a bleach solution (20% methanol, 5% acetic acid) for which a molecular weight standard for proteins (Middle Range protein Ladder, ready-to-use—Thermo Fisher Scientific, Waltham, MA, USA) was used. Electrophoresis occurred at room temperature, using a voltage of 150 V and a current of 40 mA per gel. The regions of interest were cut out from the gel manually with the aid of a sterile scalpel on a clean surface and placed in Eppendorf tubes washed with methanol and Milli-Q water. To remove the Coomassie dye, the gel bands containing the venom proteins were incubated under agitation for 10 min in a bleaching solution (50% acetonitrile; 25 mM NH_4_HCO_3_; pH 8), followed by a 10 min rest; the procedure was repeated until complete dye removal. After removing the dye, the fragments were washed with 100% acetonitrile and dried in a vacuum centrifugation system for 25 min.

During enzymatic digestion, the gels were rehydrated with trypsin solution (10–15 μg/mL trypsin; 25 mM NH_4_HCO_3_). The enzymatic solution was incubated (4 °C), the samples remained in an ice bath for 30 min, then 25 mM NH_4_HCO_3_ was added and kept at 37 °C for 20 h. After this period, the digestion tube supernatant was transferred to a methanol treated Eppendorf tube. To extract the peptides, the gel fragments were covered with a 50% acetonitrile solution; 5% TFA, and gently homogenized for 30 min, the supernatants were reduced to a volume of 5 μL in a vacuum centrifugation system and then purified in C18 ZIP TIPs micro columns following the manufacturer’s instructions.

The total venom was filtered through a 0.22-micron filter solubilized in ammonia bicarbonate (Sigma–Aldrich, St. Louis, MO, USA # A-6141) pH 8.0, containing a phosphatase inhibitor. The protein content of the samples was quantified using the BCA kit (Thermo Fisher Scientific, Waltham, MA, USA). The protein disulfide bridges were reduced by adding 10 mM DTT (Sigma–Aldrich, St. Louis, MO, USA # D-5545), followed by incubation for 30 min at 56 °C. The proteins were alkylated with 55 mM IAA (Sigma–Aldrich, St. Louis, MO, USA # I-1149), at room temperature, in the dark, for 30 min. Protein digestion was performed using trypsin (Promega # V5111), in a 1:20 ratio, at 37 °C, for 18 h. Columns C 18 (Harvard Apparatus, Holliston, MA, USA) were used for cleaning and the desalination of the sample. Digestion products were loaded onto a tandem system containing a pre-column that separates the products and passes the effluent automatically into an LTQ-Orbitrap Velos mass spectrometer (Thermo Fisher Scientific, Waltham, MA, USA). The enzymatic digestion with trypsin and desalination of the gel bands, as well as the crude venom were carried out at CEFAP: Center for research support facilities at the University of São Paulo, USP.

### 4.5. LC-MS/MS Data Analysis

After analyzing the samples by LC-MS/MS, the raw data were collected and passed through the Mascot platform (Matrix Science, Boston, MA, USA), using carbamidomethylation and methionine oxidation as variable modifications. The resulting files were exported in the .dat extension and processed in the Scaffold Q + software version 4.0 (Proteome Software, Portland, OR, USA), using as selection criteria of the presence of at least three peptide fragments, a probability rate of 95% for protein identification and a false discovery rate (FDR) of 5%. In addition, for the analysis carried out on the Scaffold Q + and Mascot platforms, a FASTA database was built containing the amino-acid sequences obtained through the predicted proteins from assembled transcripts of the transcriptome venom gland, together with sequences of possible sample contaminants such as trypsin and human keratin, to avoid alignment and coverage errors. Based on the data on the probability of protein identification and percentage of coverage, a contig for each band of venom was identified.

### 4.6. Protein Expression and Purification

The nucleotide sequence of Cryptoxin-1 was optimized for expression in *E. coli* and the construction pET24b-Cryptoxin-1 was performed using Invitrogen™ Gene Synthesis (GeneArt™ Thermo Fisher Scientific, Waltham, MA, USA).

For their expression, chemically competent *E. coli* BL21 Star™ (DE3) (Invitrogen^®^) were transformed with pET24b-Cryptoxin-1 construction or pET-42a (Novagen) containing the sequence of glutathione S-transferase (GST). For each experiment, a cell colony grown overnight from LB-agar plates was transferred into a liquid LB medium and grown overnight at 30 °C in the presence of 50 µg/mL kanamycin. This culture was diluted 1:50 into 200 mL of fresh LB broth/kanamycin. When the cell suspension reached an optical density of 0.6 at 30 °C (OD 600 nm) it was induced with a final concentration of 1 mM of isopropyl-b-D-thiogalactoside (IPTG). Cells were then grown for four hours after which the cells were collected by centrifugation at 10,000× *g* for 10 min at 10 °C (ultracentrifuge Beckman). The whole-cell pellets were then resuspended in a binding buffer (20 mM sodium phosphate pH 7.4 and 500 mM NaCl) and lysed on ice by an ultrasonication device (amplitude of 20% with 3 s pulse and 4 s interval between each pulse) for 60 s, and the process was repeated 5 times. Cell debris were removed from the protein solution by centrifugation at 10,000× *g* for 10 min in a Beckman ultracentrifuge. The entire amount of the supernatant containing the soluble protein was purified with high-performance immobilized metal affinity chromatography (IMAC) using HisTrapHP 5 mL column pre-packed (Cytiva™, Marlborough, MA, USA) coupled to the ÄKTA™ start protein purification system and then desalted into phosphate-buffered saline (PBS) buffer pH 7.4 with HiTrap^®^ Desalting Columns (Cytiva™, Marlborough, MA, USA). The endotoxin was removed with Pierce™ High-Capacity Endotoxin Removal Spin Columns (Thermo Fisher Scientific, Waltham, MA, USA) following the manufacturer’s protocol. Quantification of recombinant proteins was performed using the BCA Pierce™ Protein Assay Kit (Thermo Fisher Scientific, Waltham, MA, USA) following the manufacturer’s protocol.

### 4.7. Mass Spectrometry Analysis

Mass spectrometry of Cryptoxin-1 was performed on the MALDI-TOF Autoflex Speed (Bruker Corporation, Billerica, MA, USA) equipment following pre-established protocols for protein analysis. In summary, 0.5 μL of a saturated solution of sinapinic acid in ethanol was mixed with 100 ng of Cryptoxin-1. After drying, 1 μL of a TA30 (0.1% trifluoroacetic acid/acetonitrile in a proportion of 70/30) was added to the mixture and applied to the Ground Steel plate for analysis. Data acquisition was performed in a linear mode with positive polarity, with the following parameters: Ion Source 1—19.50 kV, Ion Source 2—17.60 kV, Lens—9.0 kV, Pulsed Ion Extraction 170 ns, Mass Range 5—70 kDa, Laser Frequency 500 Hz, Gain Detector 10.0×. The results were analyzed using the online software mMass version 5.5.0, 2013 (Martin Strohalm© Open Source Mass Spectrometry Tool).

### 4.8. Rabbit Specific Antivenom Production

The anti-venom serum from the *C. iheringi* centipede was obtained by immunization of rabbits. Two hundred micrograms of the venom and 2.5 mg of aluminum hydroxide (Brenntag Specialties, Inc., South Plainfield, NJ, USA) were added to a final volume of 1 mL of PBS. After this, 250 μL of this mixture was injected intramuscularly. After 1 month, the rabbits received five consecutive boosters of antigen with 15-day intervals. Blood was collected and sera were separated and stored at 20 °C until use. Antibodies present in the hyperimmune serum were purified on HiTrapProtein G HP 5 mL column pre-packed with high-performance protein G-Sepharose (GE Healthcare, Little Chalfont, UK) and quantified by BCA (QuantiProBCA Assay Kit, Sigma-Aldrich, St. Louis, MO, USA). To characterize the polyclonal anti-venom, ELISA and immunoblotting assays were performed. The experimental protocols were approved by the Butantan Institute Ethical Committee for Animal Research (certified by CEUAIB n° 8172250816).

### 4.9. ELISA Immunoassay

Microplates of 96-well (Sarstedt, Germany) were sensitized with 100 µL/well of the heterologous proteins in the serial 1:2 dilution from 1 to 0.008 µg/mL and incubated in a humid chamber at 4 °C for 18 h. Crude venom and GST protein were used as controls under the same conditions. Subsequently, blocking was performed with PBS containing 1% bovine serum albumin (BSA) for 30 min. After blocking, the addition of 1:200 (7.5 µg) of polyclonal IgG anti-*C. iheringi* venom antibody diluted in PBS + 1% BSA at 37 °C for 1 h. Subsequently, the microplates were incubated with a peroxidase-conjugated anti-rabbit IgG antibody (1:5000) at 37 °C for 45 min. After this, a revelation solution of OPD (ortho-phenylenediamine) was added (1 mg of OPD, 2 mL of citrate/phosphate buffer, and 1 µL of hydrogen peroxide). Then, the microplates were statically incubated, in the dark, at 24 °C for 15 min, and sulfuric acid (H_2_SO_4_) 2 N was used to stop the reaction and the plate was read in an ELISA reader (Labsystems Multiskan, Thermo Fisher Scientific, Waltham, MA, USA) at 492 nm.

### 4.10. Mice

For the experiments, BALB/c male mice (between 18 and 20 g) were bred from the animal house facilities of the Butantan Institute, São Paulo, Brazil. The animals were kept in a controlled temperature, 12/12 light/dark cycle, and were provided with standard food and water *ad libitum*. The experimental protocols were approved by the Butantan Institute Ethical Committee for Animal Research (certified by CEUAIB n° 4300061120).

### 4.11. Evaluation of Paw Edema

Mice (*n* = 6) were injected (30 μL) with Cryptoxin-1 (45 μM), GST (45 μM), or PBS (negative control) in the right hind paw. The edema-forming activity was studied after 1, 24, 48, and 72 h, by pachymeter. The results were expressed as the difference in paw thickness before (control) and after (experimental) injection (mean ± S.E.M).

### 4.12. Histological Analysis

Mice (*n* = 5) were injected in the right paw with Cryptoxin-1 45 μM/30 μL, GST 45 μM/30 μL or PBS (negative control) and 24 h after the injection, the animals were euthanized, and the right paws were collected for footpad skin removal. The samples were then fixed in 4% paraformaldehyde in PBS, pH 7.2, for 24 h. After dehydration in a crescent ethanol series up to 95%, the samples were embedded in glycol methacrylate (Leica Microsystems Nussloch GmbH, Heidelberg, Germany). Sections of 4 μm were obtained in a Microm HM340 microtome and stained with the hematoxylin-eosin solution for morphological studies of tissues.

### 4.13. Analysis of the Neutrophil Infiltrate in Footpad Tissue by Flow Cytometry

The Neutrophil migration in the footpad after 24 h of Cryptoxin-1 injection was analyzed by flow cytometry. Groups of mice (*n* = 5) were euthanized, and the right paws were removed at the tibiotarsal joint and macerated. The debris was resuspended into 1 mL of PBS and then centrifuged (5 min/1200 rpm/4 °C). The cell pellets were recovered and counted by Trypan blue exclusion (Sigma–Aldrich, St. Louis, MO, USA) using a hemocytometer. The cells that were resuspended in an RPMI-1640 cell culture media (Gibco Thermo Fisher Scientific, Waltham, MA, USA), were then incubated with anti-FcγRII/III mAb for 30 min at 4 °C. Afterward, the cell suspensions were centrifuged (5 min/1200 rpm/4 °C) and resuspended in the culture medium (10^6^ cells/well) and incubated with anti-leukocyte (CD45-APC) and anti-neutrophil (Ly6G-PE/CD11b-PeCy7) monoclonal antibodies (BD Biosciences, Franklin Lakes, NJ, USA) for 30 min at 4 °C. The cells were then washed and resuspended in PBS containing 0.1% paraformaldehyde (Merck, Darmstadt, Germany). All samples were acquired in the flow cytometer (FACS Canto II, BD Biosciences, Franklin Lakes, NJ, USA). Around 20,000 events were collected for each sample The data were analyzed using FlowJo software 7.5 (BD Biosciences, Franklin Lakes, NJ, USA). The forward and side scatter density plots (FSC × SSC) were used to exclude the debris and select the cell population, followed by the selection of the single cells. After this, the CD45^+^ cells were selected and then, using the fluorescent minus one (FMO) methodology, the CD45^+^CD11b^+^Ly6G^+^ cells were determined. The results were expressed as the mean of the percentage of CD45^+^CD11b^+^Ly6G^+^ cell population of individual mice/group ± standard error of the mean (S.E.M).

### 4.14. Statistical Analysis

All statistical analyses and graphical representations were analyzed using the GraphPad Prism 9.1.2 program. Statistical tests performed using ANOVA followed by the Bonferroni test and t Student’s test. The *p* values followed the pattern recommended by the software: * *p* < 0.05; ** *p* < 0.001; *** *p* < 0.0001; **** *p* < 0.00001.

## Figures and Tables

**Table 1:** Description of Transcriptome sequencing and Assembly of *Cryptops iheringi* and the transcriptome completeness analysis by BUSCO.

Description	Number (%)
Total Raw Paird-end Reads	15,904,398
Total High-quality Paird-end Reads	14,964,551
Total transcripts	88,774
Percent GC Content	42.76%
Transcript N50	1104
Median transcript length	416
Average transcript length	766.27
Longest transcript length	23,855
Number of transcripts >1 kb	16,266 (18.3%)
Shortest transcript length	209
Total assembled bases	68,024,656
Complete BUSCOs	885 (92.7%)
Complete and single-copy BUSCOs	713 (74.7%)
Complete and duplicated BUSCOs	172 (18%)
Fragmented BUSCOs	49 (5.1%)
Missing BUSCOs	20 (2.2%)
Total BUSCO groups searched	954 (100%)

**Table 2:** The number of transcripts from TSA/NCBI for each species from Scolopendromorpha orders and the number of hits from *C. iheringi* transcriptome assembly against the orders.

Description	Total Transcripts	Number (%)
Total *Cryptops iheringi* Hits	-	5328 (6%)
*Cryptops anomalans*	33,662	4272
*Scolopocryptops rubiginosus*	28,965	575
*Scolopendra cingulata*	23,301	283
*Scolopocryptops sexspinosus*	1540	117
*Scolopendra subspinipes*	648	32
*Scolopendra viridis*	520	29
*Hemiscolopendra marginata*	764	17
*Scolopendra morsitans*	662	3
*Scolopendra alternans*	51	0
*Scolopendra dehaani*	16,084	0

**Table 3:** Toxin identification of the major bands of *C. iheringi* venom based on the transcriptome and proteomic data. The numerical identifications correspond to the group of bands where the protein was found.

Band Group	Accession Number	Unique Peptides	Proteome Coverage	Molecular Weigth	Best Hit	Species	Identity and Acession Number
1	Ciheringi01366	32	53%	152 kDa	uncharacterized protein	*Centruroides sculpturatus*	25.07% XP_023226371.1
2	Ciheringi05450	78	77%	76 kDa	Hemocyanin	*Scolopendra dehaani*	55.99% SMH67860.1
3	Ciheringi11581	10	36%	37 kDa	No hit	-	-
3	Ciheringi14246	8	29%	27 kDa	Lipase	*Centruroides sculpturatus*	29.74% XP_023229615.1
3	Ciheringi16405	4	14%	28 kDa	Venom allergen	*Scolopendra subspinipes*	42.92% QEE04219.1
3	Ciheringi21566	5	34%	22 kDa	No hit	*-*	-
4	Ciheringi38643	12	56%	11 kDa	No hit	*-*	-
4	Ciheringi10323	4	53%	10 kDa	Lipase	*Branchiostoma floridae*	37.39% XP_035699465.1
5	Ciheringi24930	3	22%	15 kDa	No hit	*-*	-
5	Cryptoxin-1	12	68%	12 kDa	No hit	*-*	-
6	Ciheringi05125	7	74%	14 kDa	Profilin	*Orussus abietinus*	80.95% XP_012283556.1

## Data Availability

Not applicable.

## Supplementary Materials

The following are available online at https://www.mdpi.com/article/10.3390/toxins13120858/s1, Figure S1: Capillary electrophoresis of the RNA sample extracted from *C. iheringi*; Figure S2: Size distribution of *C. iheringi* cDNA library evaluated in Agilent 2100 Bioanalyzer; Table S1: *Cryptops iheringi* transcriptome data.
