# Supplementary material for: Recombinant Production and Characterization of a New Toxin from Cryptops iheringi Centipede Venom Revealed by Proteome and Transcriptome Analysis
Source: Toxins (Basel). 2021 Dec 2;13(12):858. doi: 10.3390/toxins13120858 (PMC8704451; doi:10.3390/toxins13120858)
Supplement: Supplementary file 1 [file toxins-13-00858-s001.zip › toxins-1434632-supplementary.pdf]

Supplementary Materials: Recombinant Production and Characterization of a New Toxin from *Cryptops iheringi* Centipede Venom Revealed by Proteome and Transcriptome Analysis

Lhiri Hanna De Lucca Caetano, Milton Yutaka Nishiyama-Jr, Bianca de Carvalho Lins Fernandes Távora, Ursula Castro de Oliveira, Inácio de Loiola Meirelles Junqueira-de-Azevedo, Eliana L. Faquim-Mauro [1] and Geraldo Santana Magalhães

RNA Extraction from C. iheringi Glands and cDNA Library Preparation

The quality of the material, as well as the size of the cDNA fragments, were analyzed by microcapillary gel electrophoresis in the Bioanalyzer equipment. In addition to capillary electrophoresis, the Bioanalyzer platform presented a summary of the total RNA quality of each sample, through an electropherogram (Figure 1) with fluorescence peaks per second (FU). The two peaks correspond to ribosomal RNA bands (18S and 28S) that fulfilled the quality parameters required for sequencing. As can be seen in Figure S1, the sample presented total RNA with good integrity and with two well-defined peaks, which correspond to the most abundant ribosomal units. The RNA obtained had a concentration of 902.7 ng/uL (Nanodrop, Thermo Fisher Scientific). After this evaluation, the samples were sent for the preparation of cDNA libraries.

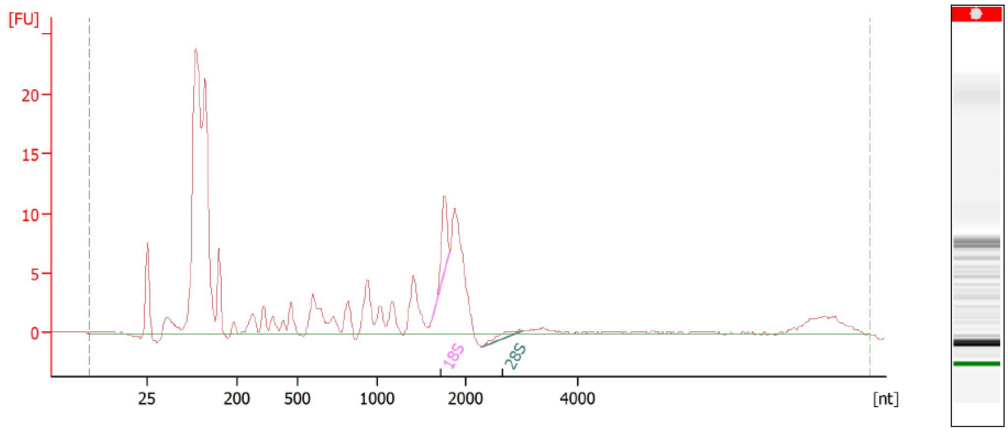

Figure S1. Capillary electrophoresis of the RNA sample extracted from *C. iheringi.*

After the construction of the cDNA library, the quality of the material, as well as the size of the cDNAs fragments were analyzed by microcapillary gel electrophoresis in the Bioanalyzer equipment (Figure S2). The results indicated that the cDNA was of good quality.

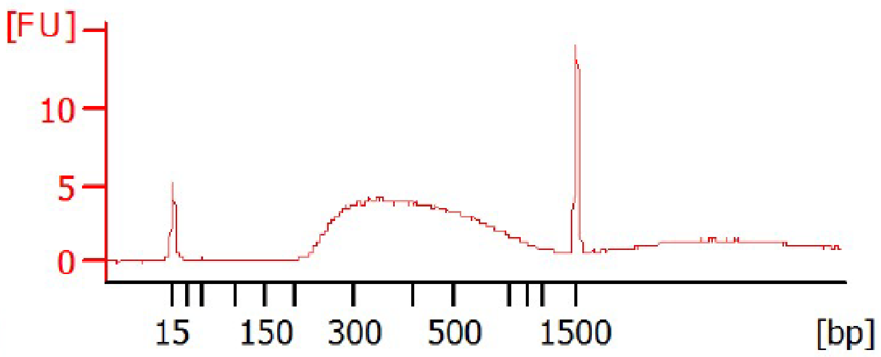

Figure S2. Size distribution of *C. iheringi* cDNA library evaluated in Agilent 2100 Bioanalyzer. The preparation was free of primer dimers and short fragments (< 300 bp), with the peak size at 1.5 kb.
